# Proteomic identification of cyclophilin A as a potential biomarker and therapeutic target in oral submucous fibrosis

**DOI:** 10.18632/oncotarget.11254

**Published:** 2016-08-12

**Authors:** Yao Yuan, Xiaohui Hou, Hui Feng, Rui Liu, Hao Xu, Wang Gong, Jing Deng, Chongkui Sun, Yijun Gao, Jieying Peng, Yingfang Wu, Jiang Li, Changyun Fang, Qianming Chen

**Affiliations:** ^1^ State Key Laboratory of Oral Diseases, West China Hospital of Stomatology, Sichuan University, Chengdu China, 610041; ^2^ Department of Endodontics, School & Hospital of Stomatology, Shanghai Engineering Research Center of Tooth Restoration and Regeneration, Tongji University, Shanghai China, 200072; ^3^ Xiangya Stomatological Hospital, Central South University, Changsha, China, 410008; ^4^ Department of Stomatology, Second Xiangya Hospital, Central South University, Changsha, China, 410008; ^5^ Center of Stomatology, Xiangya Hospital, Central South University, Changsha, China, 410008

**Keywords:** CypA, proteomics, oral submucous fibrosis, fibroblast

## Abstract

Oral submucous fibrosis (OSF) is a pre-cancerous lesion, which is characterized by fibrosis of the oral submucosa. Despite large body of studies focusing on this disease, the molecular mechanisms underlying the progression of OSF remained unclear. In this study, 2-DE-based proteomic approaches were employed to identify the differently expressed proteins between OSF and normal tissues. In total, 88 proteins were identified with altered expression levels, including CypA. Upregulation of CypA was further validated through immunohistochemistry staining combined with Q-PCR and western blot by using clinical samples. Statistical analyses reveal that CypA expression level is correlated to the progression of OSF. Finally, functional study reveals a pro-proliferative property of CypA in fibroblast cells by using multiple *in vitro* models. The present data suggest that CypA might be a potential biomarker and therapeutic target for OSF, and will lead to a better understanding of OSF pathogenesis.

## INTRODUCTION

Oral submucous fibrosis (OSF) is a chronic pre-cancerous oral lesion that mainly occurs in Asia. Most OSF patients suffered from spicy sensitivity, difficulties in mouth opening or tough movement, and stiffness of oral mucous [1]. A great proportion of patients also suffered from hypogeusia, thirsty, numbness and mucosal blister. More importantly, parts of OSF patients may finally develop into oral carcinoma. Epidemiological study has shown that the malignant transformation rate of OSF patients is 2%-12% [2]. Development of OSF was related to several factors, including an unhealthy diet, nutrient imbalance, immune hyper-activation and genome instability [3]. It has been noted that the incidence of OSF in those areas where people have the habit of chewing areca nut is markedly higher than other regions [4]. The main harmful chemical in the areca nut, arecoline, can induce cytotoxicity in human buccal mucosa and causes the fibrosis lesion [5]. In spite of a large body of studies focusing on the etiology and pathogenesis of OSF, the molecular mechanisms of this disease are still not fully understood.

Aberrantly enhanced fibroblast proliferation results in accumulation of fibroblast extra cellular components, and virtually causes tissue dysfunction. Molecular factors that function in regulating fibroblast proliferation are considered to play an important role in OSF development [6]. It is reported that increased expression of pro-fibrotic mediators, such as: connective tissue growth factor (CTGF), collagen I, platelet derived growth factor B (PDGF B), transforming growth factor β (TGFβ), induced excessive fibroblast proliferation and collagen synthesis, and finally leads to fibrosis [7, 8]. It is also reported that relaxin treatment could effectively inhibit the proliferation of fibroblasts and reduce the collagen accumulation, which was considered as an approach to alleviate myocardial fibrosis disease [9]. It is also shown that lysophosphatidic acid induced fibrosis via enhancing the proliferation of fibroblasts [10]. Moreover, alveolar epithelial cells were capable to promote fibroblast proliferation and finally induce fibrosis via producing FGF-2 [11].

Nowadays, it has become apparent that some molecular mechanisms cannot be studied only at the DNA or RNA level. Proteomics provided a quick and high-throughput approach to study the translation, intracellular localization and post-translational modification of all the proteins. Proteomics has gradually become an powerful strategy to screen the biomarker and drug target in various diseases [12]. By proteomic strategies, clusters of valuable biomarkers in fibrosis have been identified, such as CFTR in cystic fibrosis lung disease [13] and calgranulin B in idiopathic pulmonary fibrosis disease [14]. In this study, 2-DE-based proteomics approach was used to identify the differentially expressed proteins in OSF. We demonstrate that expression of CYPA was up-regulated in OSF tissues versus normal tissues. Further, functional study shows that CYPA plays a crucial role in regulating fibroblast cells proliferation.

## RESULTS

### Proteomic profiling of the differently expressed proteins in OSF

**Figure 1:**
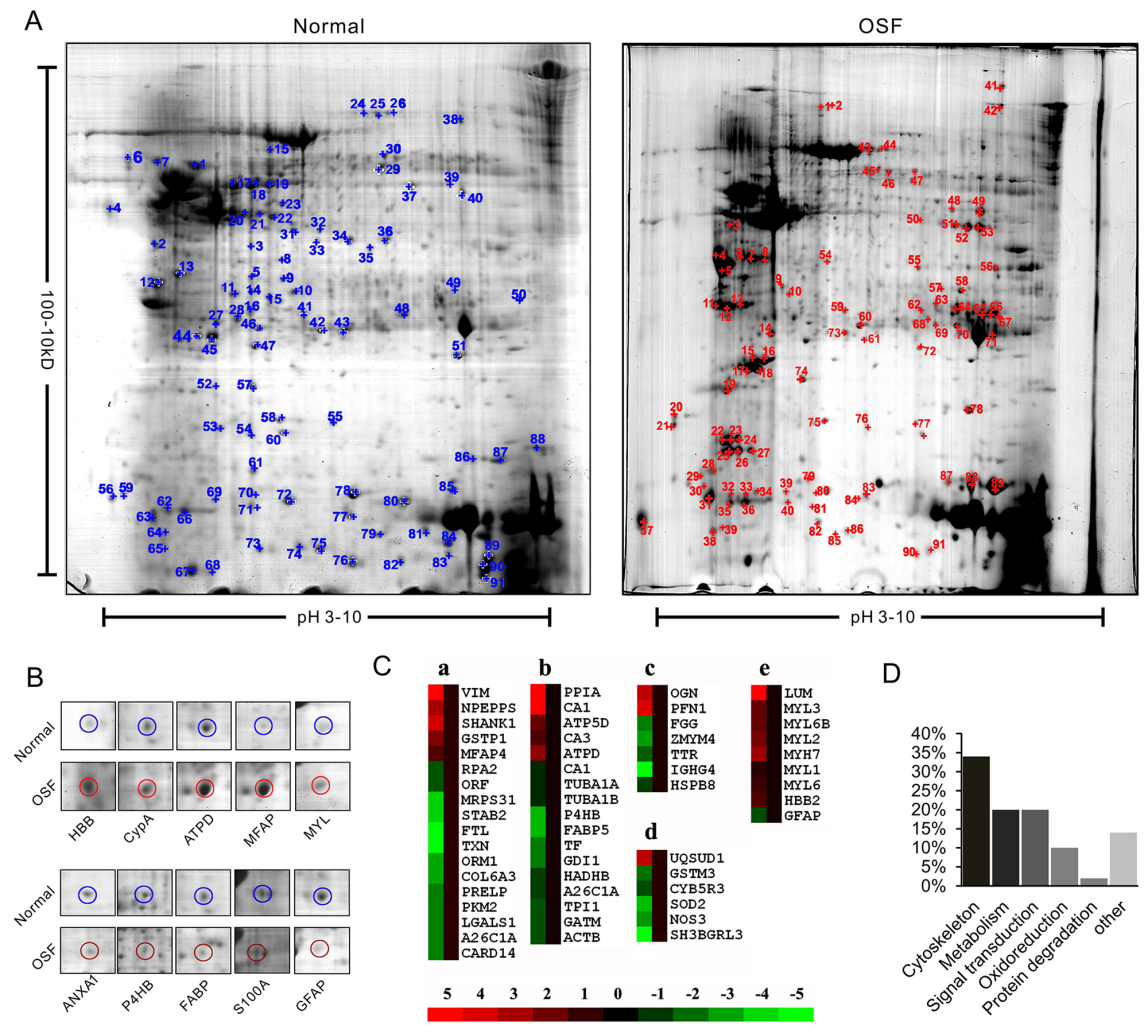
Proteomic identification of differently expressed proteins between normal and OSF tissue **A.** Representative 2-DE gel images of human normal and OSF tissues. The protein extracts were separated on the pH 3-10 nonlinear IPG strips and followed with 12% SDS-PAGE in the second dimension, then visualized by CBB staining. 2-D gel images were analyzed by PDQuest software and the protein spots were labeled with numbers. **B.** Representative spots with changed density. **C.** Protein cluster analysis was performed by using Cluster software. Expression of proteins in the normal tissues was constantly set as 0. Meanwhile proteins up-regulated and down-regulated in the OSF tissue are in red and green, respectively. The intensity of the color red or green is correspondent to the level of differentiation according to the color strip. **D.** 88 identified proteins were classified into six groups according to their function.

2-DE profiling was employed to analyze the differently expressed proteins between normal and OSF tissues. As shown in Figure 1A, around 1000 protein spots were found in each gel, and most of these spots were located within the range of pH3-8 and molecular weight 10-60kD. The intensity of each gel spot was quantified as the percentage of the total intensity of all spots. Differentially expressed proteins were defined according to two factors: (1) the gel spots were emerged at least three times in all the five parallel 2-DE experiments; (2) the intensities of the spots were changed at least two fold. In total, 91 spots were significantly changed intensities were finally identified by PDQuest software (Figure 1A). Ten representative spots and their surrounding areas were shown in Figure 1B.

To identify the protein according to each spot, gel spots were digested and subsequently subjected to MS/MS analysis. The raw MS/MS data of each spot was analyzed by online engine MASCOT (http://www.matrixscience.com). The detail information, including pI, molecular weight, and the MOWSE score of the 36 up-regulated and 52 down-regulated proteins was listed in Table 1 and Table 2. Among them, CypA showed notable upregulation in OSF tissues. Representative MS/MS data according to CypA-derived peptide ‘SIYGEKFEDE’ was shown in Supplementary Figure S1. The changes in the expression level of each protein were then analyzed by Cluster software (Figure 1C), and the identified proteins were divided into six groups according to their functions (Figure 1D).

**Table 1:** The up-regulated proteins identified by mass spectrum

Accession No. ^a^	Protein name ^b^	Gene name	MW/PI ^c^	No. of Pep	Score ^d^
**Cytoskeleton**					
P02768	Serum albumin	ALB	69367/5.67	40	685
P08865	40S ribosomal protein SA	RPSA	32,854/4.79	8	82
P51884	Lumican	LUM	38,429/ 6.17	3	69
P02533	Keratin, type I cytoskeletal 14	KRT14	51,622/5.09	5	104
P04264	Keratin, type II cytoskeletal 1	KRT1	66,018/ 8.16	2	63
P35527	Keratin, type I cytoskeletal 9	KRT9	62,129/ 5.19	2	79
P19013	Keratin, type II cytoskeletal 4	KRT4	57,285/6.25	2	73
P13647	Keratin, type II cytoskeletal 5	KRT5	62,378/7.58	3	75
P05787	Keratin, type II cytoskeletal 8	KRT8	53,704/5.52	2	65
P05976	MLC1F	MYL1	21,145/4.97	2	78
P08590	Myosin light chain 3	MYL3	21,932/5.03	4	116
P14649	Myosin light chain 6B	MYL6B	22,764/5.56	4	254
P60660	Myosin light polypeptide 6	MYL6	16,930/4.56	2	81
P10916	MLC-2	MYL2	18,789/ 4.92	3	74
P12883	Myosin-7	MYH7	223,097/5.63	3	65
P68871	Hemoglobin subunit beta	HBB	15,998/ 6.81	30	469
O93349	Hemoglobin subunit beta	HBB2_TRENE	16,326/6.03	5	124
P07355	Annexin A2	ANXA2	38,604/ 7.56	10	104
**Metabolism**					
P23542	Aspartate aminotransferase, cytoplasmic	AAT2	46,058/ 8.45	3	93
P62937	PPIase A	PPIA	18,012/7.82	16	307
P30049	F-ATPase delta subunit	ATP5D	17,490/4.53	2	69
P00915	Carbonic anhydrase 1	CA1	28,870/ 6.63	2	120
P07451	Carbonic anhydrase 3	CA3	29,557/6.94	6	185
P30049	ATP synthase subunit delta, mitochondrial precursor	ATPD	17489.9/5.34	11	168
**Signal transduction**					
P08670	Vimentin	VIM	53,652/5.06	4	120
P13929	Beta-enolase	ENO3	46,987/7.73	2	78
P09211	Glutathione S-transferase P	GSTP1	23,356/5.44	3	69
P55786	PSA	NPEPPS	103,276/5.49	2	64
P55083	Microfibril-associated glycoprotein 4	MFAP4	28,648/ 5.21	7	178
Q9Y566	Shank1	SHANK1	225,021/8.27	3	69
**Oxidoreduction**					
Q9P0J0	NADH dehydrogenase (ubiquinone) 1 alpha subcomplex,	NDUFA13	16687.6/8.02	2	129
A7MCR1	Ubiquinol-cytochrome c reductase, Rieske iron-sulfur polypeptide 1	UQCRFS1	29,714/273	4	221
**Other**					
P69905	Hemoglobin subunit alpha	HBA1	15126/8.73	15	471
P14649	smooth muscle and non-muscle myosin alkali light chain 6B	MYL6B	22763.9/5.56	19	377
P20774	Mimecan	OGN	33,922/5.22	3	92
P07737	Profilin-1	PFN1	15,054/8.47	2	83

a Accession numbers were obtained from the ExPASy database.

b For some proteins, a few isoforms were identified as the same protein.

c The theoretical molecular weight (Da) and pI from the ExPASy database.

d The protein scores based on the MOWSE.

**Table 2:** The down-regulated proteins identified by mass spectrum

Accession no. ^a^	protein name ^b^	Gene name	MW/PI ^c^	No. of Pep	Score ^d^
**Cytoskeleton**					
P14136	Glial fibrillary acidic protein	GFAP	49,880/5.42	5	89
Q6RHW0	CK-9	Krt9	72,527/ 5.55	2	84
Q86Y46	Keratin, type II cytoskeletal 73	KRT73	58,923/6.93	2	63
Q7RTS7	Keratin, type II cytoskeletal 74	KRT74	57,865/7.59	3	75
O95678	Keratin, type II cytoskeletal 75	KRT75	59,504/ 7.60	8	91
**Calcium binding protein**					
P62158	Calmodulin	CALM1	16,838/ 4.09	2	87
P06703	Protein S100-A6	S100A6	10,180/5.32	13	526
P06702	Protein S100-A9	S100A9	13,242/ 5.71	24	295
P31949	Protein S100-A11	S100A11	11,740/6.56	6	87
P04083	Annexin A1	ANXA1	38,714/6.64	9	258
P09525	Annexin A4	ANXA4	35,883/ 5.85	5	112
P50995	Annexin A11	ANXA11	54,390/7.53	2	63
**Metabolism**					
P07237	PDI	P4HB	57,116/4.69	19	573
P55084	TP-beta	HADHB	51,294/9.24	3	79
P00915	Carbonic anhydrase 1	CA1	28,870/6.63	18	254
P50440	Glycine amidinotransferase, mitochondrial	GATM	48,455/ 6.43	2	76
Q71U36	Tubulin alpha-1A chain	TUBA1A	50,136/4.94	3	87
P68363	Tubulin alpha-1B chain	TUBA1B	50,152/4.94	2	88
P60174	TIM	TPI1	26,669/6.51	4	132
P02787	Serotransferrin	TF	77,050/6.70	13	172
Q01469	Fatty acid-binding protein	FABP5	15,164/ 6.82	3	98
P31150	Rab GDI alpha	GDI1	50,583/ 5.00	2	72
P60709	Actin, cytoplasmic 1	ACTB	41,737/ 5.29	11	309
Q6S8J3	ANKRD26-like family C member 1A	A26C1A	121,363/5.83	3	69
**Signal transduction**					
P51888	Prolargin	PRELP	43,810/9.45	2	83
P02763	AGP 1	ORM1	23,512/ 5.00	5	94
Q92665	S31mt	MRPS31	45,318/8.31	2	86
P02792	Ferritin light chain	FTL	20,020/5.51	2	54
P14618	CTHBP	PKM2	57,937/7.95	4	76
P15927	RP-A p32	RPA2	29,247/ 5.75	3	87
Q8WWQ8	Stabilin-2	STAB2	276,988/5.99	3	65
P10599	Thioredoxin	TXN	11,737/4.82	4	82
Q9BXL6	Carma 2	CARD14	113,300/ 5.62	2	67
P12111	Collagen alpha-3(VI) chain	COL6A3	343,665/6.15	2	84
Q6S8J3	ANKRD26-like family C member 1A	A26C1A	121,363/5.83	3	97
P09382	Galectin-1	LGALS1	14,716/5.34	6	205
**Protein degradation**					
Q9UL46	Proteasome activator complex subunit 2	PSME2	27,362/ 5.44	4	113
P01009	Alpha-1-antitrypsin	SERPINA1	46,737/5.37	3	152
**Oxidoreduction**					
P00441	Superoxide dismutase [Cu-Zn]	SOD1	15,936/5.70	3	79
P04179	Superoxide dismutase [Mn], mitochondrial	SOD2	24,722/6.86	21	759
P21266	Glutathione S-transferase Mu 3	GSTM3	26,560/ 5.37	2	86
P00387	B5R	CYB5R3	34,235/6.87	2	63
P29474	Nitric oxide synthase, endothelial	NOS3	133,289/6.98	3	125
Q9H299	SH3BP-1	SH3BGRL3	10,438/4.82	2	78
Q9H299	SH3BP-1	SH3BGRL3	10,438/4.82	5	157
**Other**					
P01861	Ig gamma-4 chain C region	IGHG4	35,941/ 8.92	3	94
P02675	Fibrinogen beta chain	FGB	55,928/4.14	2	87
P02679	Fibrinogen gamma chain	FGG	51,512/5.24	5	112
Q5VZL5	Zinc finger MYM-type protein 4	ZMYM4	172,788/6.46	3	85
Q9UJY1	Heat shock protein beta-8	HSPB8	21,604/	3	69
P02766	Transthyretin	TTR	15,887/5.35	5	188
P06732	muscle creatine kinase M	CKM	43101.12/6.77	28	592

a Accession numbers were obtained from the ExPASy database.

b For some proteins, a few isoforms were identified as the same protein.

c The theoretical molecular weight (Da) and pI from the ExPASy database.

d The protein scores based on the MOWSE.

### Bioinformatics analyses of the identified proteins

**Figure 2:**
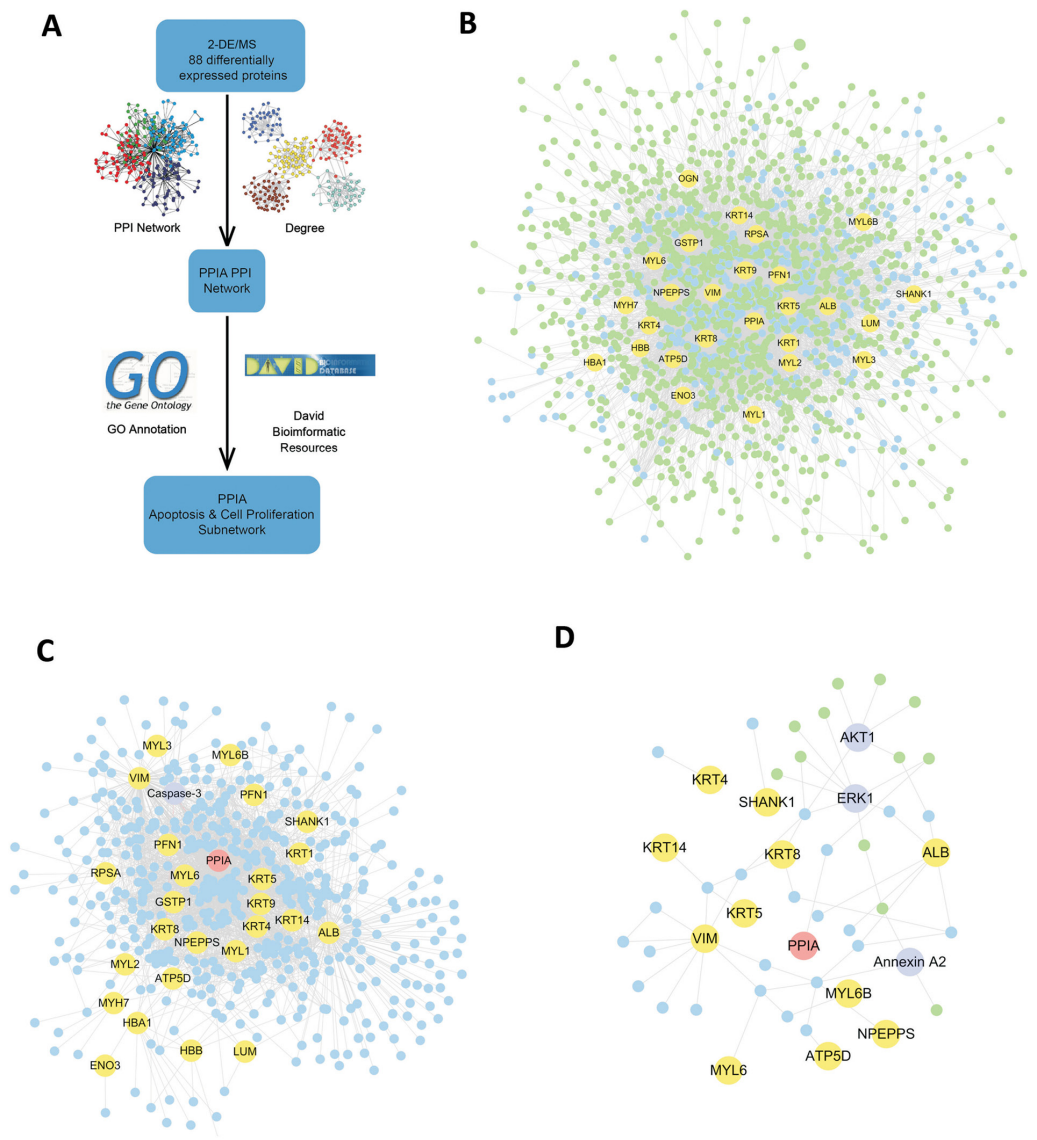
Bioinformatics analysis of the identified proteins **A.** The general workflow of bioinformatics analysis. **B.** Total PPI network which was generated based on the 88 identified proteins. **C.** The predicted CypA-associated proteins involved in apoptosis. **D.** The predicted CypA associated proteins involved in cell proliferation.

To explore the potential function of the identified proteins, Protein-Protein Interaction (PPI) network and functional annotation was performed (Figure 2A). The PPI pairs that were related to the identified proteins were extracted from online PrePPI database. As results, a total of 13454 pairs of PPIs were addressed (Figure 2B). The PPI-network was then analyzed via GO Annotation. Strikingly, clusters of proteins functioning in the regulation of cell proliferation and apoptosis were found in the PPI network of CypA. Considering that the excessive proliferation of fibroblasts was closely related to the progression of OSF, CypA was selected for further study.

### CypA is up-regulated in OSF

**Figure 3:**
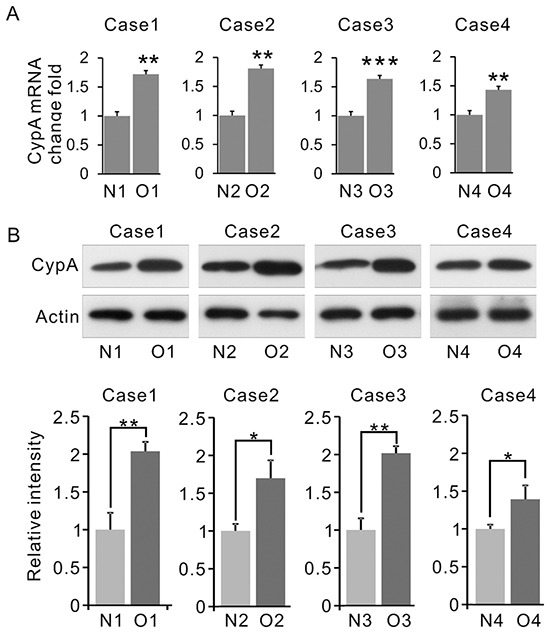
Expression of CypA is up-regulated in OSF **A.** Expression of CYPA in OSF and normal tissues was examined by Q-PCR. The total mRNA was normalized by β-actin. **B.** Expression of CYPA in OSF and normal tissues was examined by western blot. β-actin was used as an internal control. All data were representative of at least three independent experiments. ***, P<0.001; **, P<0.01; *, P<0.05.

To validate the aforementioned proteomic data, expression of CypA in clinical samples were examined. As shown in Figure 3A, the average level of CypA mRNA was increased in OSF tissues versus normal counterparts. Similarly, by western blots, OSF tissues also showed a markedly increased CypA expression (Figure 3B). These data suggested that CypA expression was elevated at both transcriptional and translational levels, which is consistent with our proteomic identifications.

### CypA expression level is correlated with OSF progression

**Figure 4:**
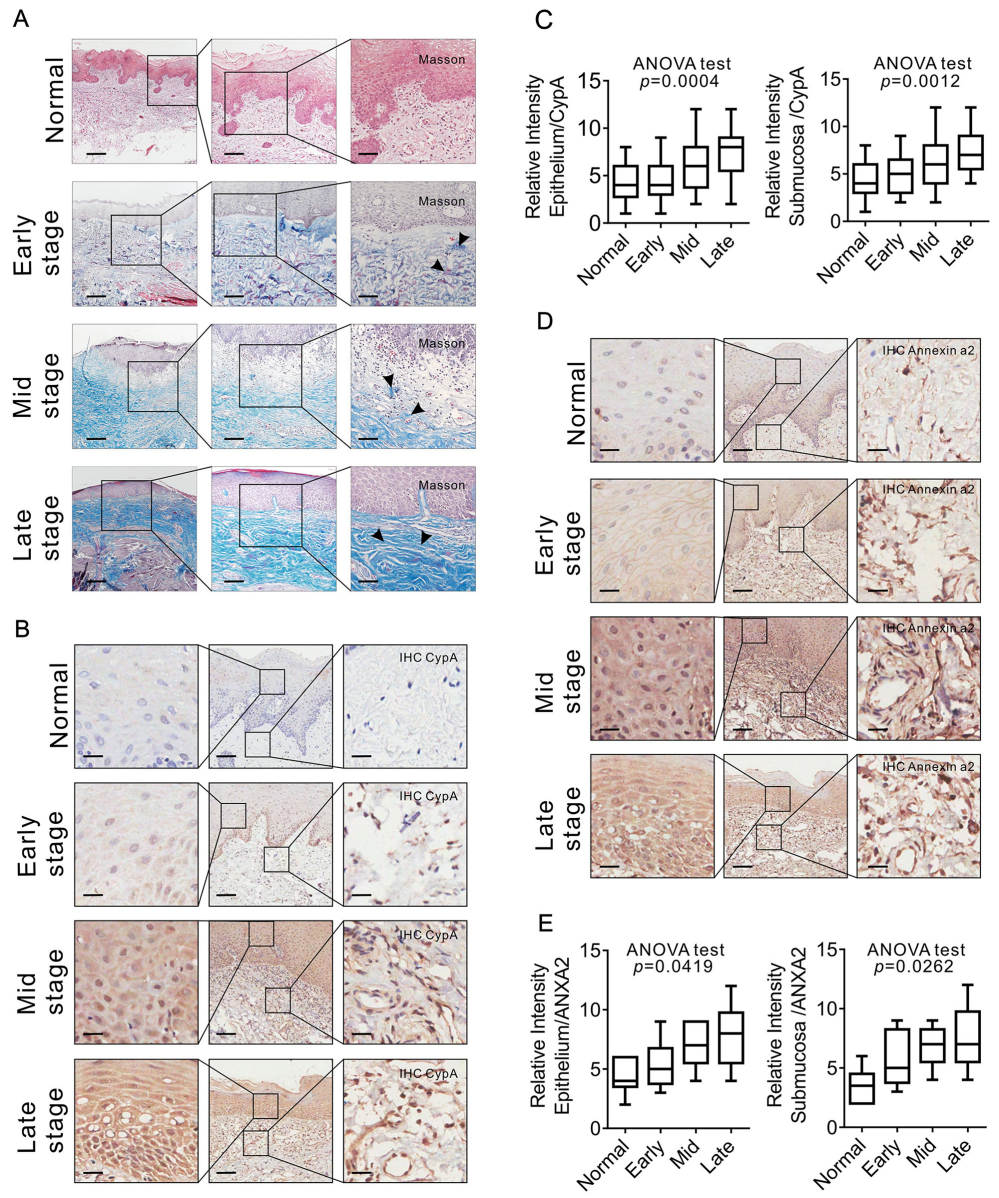
CYPA expression is associated with OSF progression **A.** Collagen deposition in OSF tissues at different stages was examined by Masson staining. 20 cases of normal tissues, 20 cases of early stage OSF tissues, 20 cases of mid stage OSF tissues and 20 cases of late stage OSF tissues were used. Scale bar: left panels, 500 μm; mid panels, 200μm; right panels, 100 μm. **B.** Expression of CYPA in OSF tissues at different stages was examined by IHC staining. 20 cases of normal tissues, 20 cases of early stage OSF tissues, 20 cases of mid stage OSF tissues and 20 cases of late stage OSF tissues were used. Scale bar: left panels, 100 μm; mid panels, 500 μm; right panels, 100 μm. **C.** Expression of CYPA in OSF tissues at different stages was scored and compared. **D.** Expression of Annexin A2 in OSF tissues at different stages was examined by IHC staining. 15 cases of normal tissues, 15 cases of early stage OSF tissues, 15 cases of mid stage OSF tissues and 15 cases of late stage OSF tissues were used. Scale bar: left panels, 100 μm; mid panels, 500 μm; right panels, 100 μm. **E.** Expression of Annexin A2 in OSF tissues at different stages was scored and compared.

To analyze the relationship between CypA expression and OSF progression, IHC staining was performed by using clinical OSF samples at different stages. As a pilot study, Masson staining showed a severe collagen deposition in those OSF tissues at later stages compared to early stages, which was in line with previous reports (Figure 4A). As shown in Figure 4B-4C, relatively weak CypA signal was detected in both epithelial and submucosa areas from normal and early-stage OSF tissues, while an intense CypA immunoreactivity was observed in both epithelial and submucosa areas from those OSF tissues at mid and late stages (epithelium F=6.734 *p*=0.0004; submucosa F=5.819 *p*=0.0012).

Since Annexin A2 was identified in the CypA-associated PPI network, of our particular interest, expression of Annexin A2 at different OSF stages was examined. As shown in Figure 4D-4E, an marked increasing expression tendency of Annexin A2 was observed in either epithelial or submucosa area from normal tissue, early stage tissues to mid and late stage tissues, which was similar to the expression pattern of CypA (ANOVA test, epithelium F=3.288 *p*=0.0419; submucosa F=3.805 *p*=0.0262). To analyze the potential correlations between the expression of CypA and Annexin A2, Pearson correlation test was used to construct the correlation model. Statistical results revealed that the expression level of CypA was positively correlated with Annexin A2, and the Pearson correlation coefficient is 0.569 (*p*=0.004). These results suggest that CypA expression level is correlated with OSF progression.

### CypA expression is correlated with the malignant transformation of OSF

**Figure 5:**
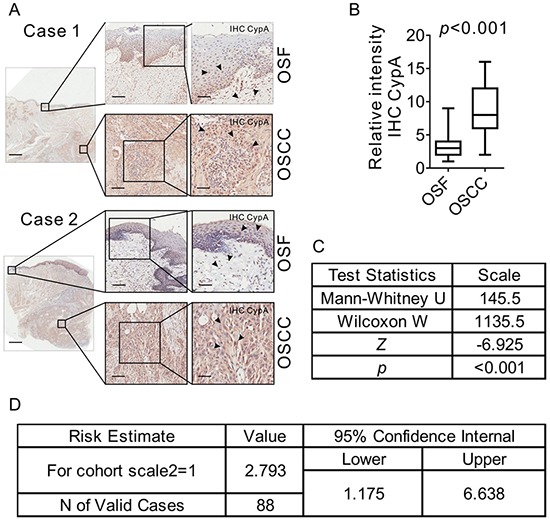
CypA is up-regulated in OSCC arising from OSF **A.** 44 OSCC tissues with its surrounding OSF tissues were collected. Expression of CYPA in both OSF and OSCC areas was examined by IHC staining. Scale bar: left panels, 2 mm; mid panels, 500 μm; right panels, 100 μm. **B.** Expression of CYPA in either OSF or OSCC areas were scored and compared. **C.** Mann-Whitney was used to analyze the potential correlations between the CypA expression level and malignant transformation of OSF. **D.** Odds ratio analysis was used to analyze the risks of malignant transformation of OSF with CypA expression level.

OSF has long been considered as a pre-cancerous state, with a malignant transformation rate of 2%-12% [3]. To investigate the potential role of CypA in the malignant transformation of OSF, expression of CypA was examined in OSCC tissues that containing both tumor area and the surrounding OSF lesions. As shown in Figure 5A, a bulky increased CypA expression was observed in tumor areas compared the adjacent OSF areas. Up-regulation of CypA in tumor cells were confirmed by either t-test (*p*<0.001, Figure 5B) or Mann-Whitney test (*Z* statistic= -6.925, *P*<0.001, Figure 5C). Further, Spearman correlation study was used to analyze the potential correlations between CypA expression and malignant transformation rate of OSF. As shown in Figure 5D, CypA expression level was positively correlated with the malignant transformation of OSF (Spearman correlation study, *rho*=0.908, *P*<0.001), and OSF patients with high CypA expression were more risky for malignant transform (*odds ratio=*2.793, 95CI%=1.175). These results suggested that CypA expression is correlated with the malignant transformation of OSF.

### Modulation of CypA expression regulates fibroblast cell proliferation

**Figure 6:**
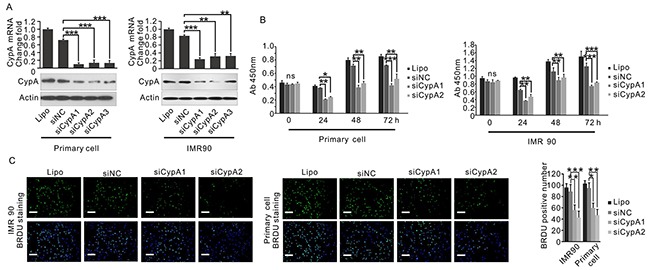
Loss of CypA inhibits fibroblast cell proliferation **A.** IMR90 and primary oral fibroblast were transfected with three distinct CypA siRNAs. Expression of CypA was examined by Q-PCR and western blot. **B.** IMR90 and primary oral fibroblast were transfected with siCypA1, siCypA2 or siNC, respectively. Proliferation of IMR90 and primary oral fibroblast cells was examined by CCK8 assay. **C.** IMR90 and primary oral fibroblast were transfected with siCypA1, siCypA2 or siNC, respectively. Proliferation of IMR90 and primary oral fibroblast cells was examined by BrdU assay. All data were representative of at least three independent experiments. ***, P<0.001; **, P<0.01; *, P<0.05.

It is well accepted that excessive proliferation of fibroblasts is one of the major features of OSF [15, 16]. To explore the role of CypA in the regulating fibroblast cell viability, human primary oral fibroblast cell and human fibroblast cell line IMR90 used as *in vitro* models. As a pilot study, both primary oral fibroblast cells and IMR90 cells were transfected with three distinct siRNAs targeting CypA. As results, siCypA1 and siCypA2 showed a better knockdown efficiency in these cells at both mRNA and protein levels (Figure 6A). Therefore, siCypA1 and siCypA2 were selected for further study. As shown in Figure 6B-6C, knockdown of CypA by siCypA1 substantially reduced proliferation rate in primary oral fibroblast cells and IMR90 cells, by both CCK8 assay and BrdU assay. Similar results were observed when the cells were treated with siCypA2 (Figure 6B-6C), which ruled out the possibility of off-target effects. To further determine the impact of CypA expression fibroblast proliferation, primary oral fibroblast cells and IMR90 cells were transfected with CypA expression vector, which resulted in notable expression of exogenous CypA (Supplementary Figure S2A). As expected, those fibroblast cells with high CypA expression showed an apparently enhanced cell growth compared to control cells (Supplementary Figure S2B-C). These results suggested a pro-proliferative role in regulating fibroblast viability.

### Loss of CypA induces apoptosis in human fibroblasts

**Figure 7:**
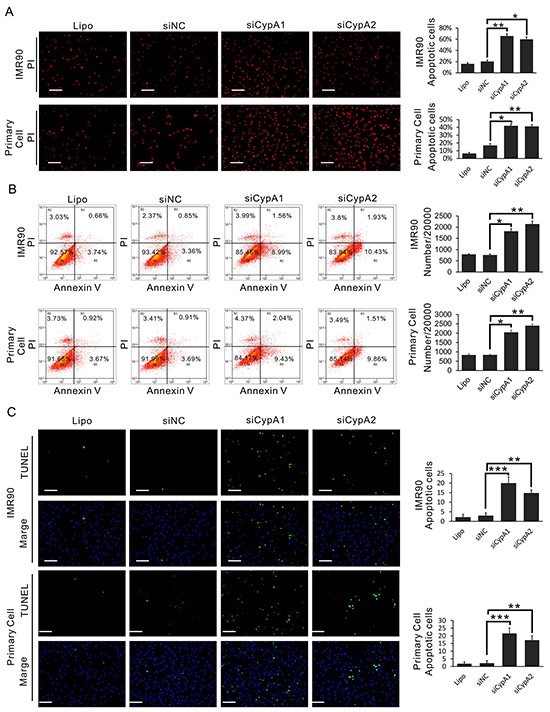
Loss of CypA induces apoptosis in fibroblast cells **A.** IMR90 and primary oral fibroblast were transfected with siCypA1, siCypA2 or siNC, respectively. Cell death of IMR90 and primary oral fibroblast cells was examined by PI staining. **B.** IMR90 and primary oral fibroblast were transfected with siCypA1, siCypA2 or siNC, respectively. Cell death of IMR90 and primary oral fibroblast cells was examined by PI/Annexin V staining-based flow cytometry assay. **C.** IMR90 and primary oral fibroblast were transfected with siCypA1, siCypA2 or siNC, respectively. Cell death of IMR90 and primary oral fibroblast cells was examined by TUNEL assay. All data were representative of at least three independent experiments. ***, P<0.001; **, P<0.01; *, P<0.05.

Since a cluster of apoptotic proteins were found in the CypA-associated PPI network, we asked whether CypA was involved in the regulation of fibroblast cell death. As shown in Figure 7A, siRNAs-mediated knockdown of CypA induced significant cell membrane permeability to phenylphenanthridinium diiodide (PI), suggesting an increased cell death upon loss of CypA. Since PI permeability could be also observed in necrotic cell, exposure of Annexin V and breaks of genomic DNA, both of which are specific markers for apoptotic cells, were examined [17]. As results, flow cytometry assay showed that silencing of CypA induced apparently more PI/Annexin V-double positive cells versus control groups (Figure 7B). Accordingly, DNA breaks which are labeled with fluorescent probes, were markedly accumulated in siCypA-treated cells, revealed by TUNEL assay (Figure 7C). These data suggested that loss of CypA induces apoptosis in human fibroblasts.

### CypA regulates ATK, ERK and Caspase 3, and reduces mitochondrial membrane potential

**Figure 8:**
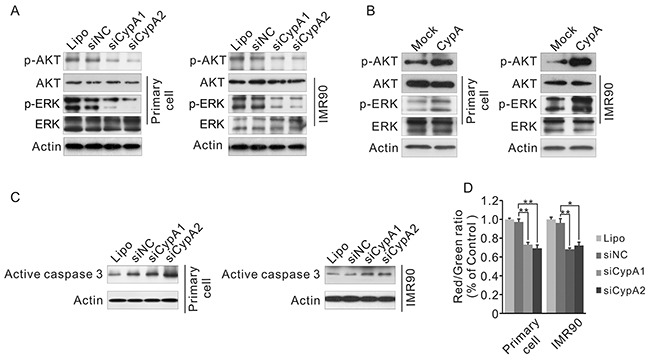
Loss of CypA activates AKT, ERK and Caspase 3, and reduces the mitochondrial membrane potential **A.** IMR90 and primary oral fibroblast were transfected with transfected siCypA1, siCypA2 or siNC, phosphorylation of AKT and ERK was examined by western blot. **B.** IMR90 and primary oral fibroblast were transfected with transfected mock or CypA expression vector, phosphorylation of AKT and ERK was examined by western blot. **C.** IMR90 and primary oral fibroblast were transfected with transfected siCypA1, siCypA2 or siNC, activation of Caspase 3 was examined by western blot. **D.** IMR90 and primary oral fibroblast were transfected with transfected siCypA1, siCypA2 or siNC, mitochondrial membrane potential was examined by JC-1 staining. All data were representative of at least three independent experiments. ***, P<0.001; **, P<0.01; *, P<0.05.

To explore the potential mechanisms underlying CypA-mediated regulation on fibroblast viability, activation status of ATK, ERK and Caspase 3 was examined, since these protein factors were identified in the CypA-associated PPI network (Figure 2C-2D). As shown in Figure 8A-8C, knockdown of CypA, substantially reduced the phosphorylated form of both AKT and ERK, and induced the active form of Caspase 3 in fibroblast cells. Accordingly, though no changes were found in Caspase 3 activation (data not shown), phosphorylation of AKT and ERK was markedly enhanced in CypA overexpressed cell compare to control cells (Figure 8B).

Mitochondrial disorder has been shown to be implicated in the induction of apoptosis. Increased mitochondrial permeability leads to dysregulation of oxidative phosphorylation, release of pro-apoptotic factors as well as loss of mitochondrial membrane potential [18]. To investigate whether mitochondrial disorder was involved in CypA knockdown-induced apoptosis, mitochondrial membrane potential was tested by JC-1 staining. As shown in Figure 8D, mitochondrial membrane potential was lower in siCypA-treated cell, indicated by the reduced ratio of red and green fluorescent signals. These results suggested that mitochondrial disorder might play a role in fibroblast apoptosis upon loss of CypA.

## DISCUSSION

Oral submucous fibrosis (OSF) is a latent and chronic fibrosis disease, and the morbidity was increased every year. It has been widely known that OSF is a pro-cancerous state, and 2%-12% of OSF patients may finally transform into OSCC [2, 19, 20]. However, the molecular mechanisms of OSF development remain unclear. In this study, 2-DE-based proteomics strategy was used to profile the differently expressed proteins in OSF. As a result, 84 distinct proteins with altered expression level (32 up-regulated and 52 down-regulated) were identified. Most of these proteins were involved in metabolism (20%), cytoskeleton (34%), signal transduction (20%) and oxidoreduction (10%).

Among these proteins, a cluster of keratin family members were identified to be up-regulated in OSF tissues, and some of them were documented to be related with fibrosis, such as: KRT14 [21], KRT5 [22], and KRT8 [23]. KRT14 and KRT8 was previously found to be overexpressed in cystic fibrosis at both RNA and protein level [24]. Simultaneous expression of KRT14 and TGF-beta1 induced proliferation in dermal fibroblast cells and promoted the wound healing [21]. Identification of upregulation of KRT14, KRT5 and KRT8 in present data suggests that upregulation of these KRTs may play a role in OSF development.

The Annexin family members, such as ANXA1 [25] and ANXA2 [26], were considered as pro-fibrosis factors. It is reported that the serum level of ANXA2 was found to be elevated in liver fibrosis cases companied with chronic hepatitis B virus, and was considered as a candidate bio-marker for liver fibrosis [27]. In our data, we demonstrate that expression of Annexin A2 was enhanced in later stages of OSF versus early stage, which was positively associated with CypA expression, suggesting that CypA and Annexin A2 may synergistically regulates OSF development.

Increasing studies have demonstrated the links between excessive reactive oxygen species (ROS) and OSF development [28]. It is shown that arecoline triggered ROS generation via modulating Transglutaminase-2, which is a key step during arecoline-mediated OSF [29]. In this study, expression of two anti-oxidant proteins, SOD1 and SOD2, was downregulated in OSF by our proteomic screening. Both of SOD1 and SOD2 play important roles in maintaining redox balance by scavenging intracellular ROS [30]. Considering the previous report that expression of CypA could be induced under oxidative stress [31, 32], it is reasonable to infer that loss of SOD1 and SOD2 as well as the subsequent accumulation of cellular ROS might be a upstream events of CypA upregulation during OSF development.

CypA, which belongs to the cyclophilins family with peptidyl-prolyl isomerase enzymatic activity, was involved in cellular metabolism and energy homeostasis. As the mostly abundant cyclophilin, this 18-kDa protein was closely related with proteins folding [33–35]. The abnormal expression level of CypA was documented during fibrosis progression. It is has been reported that, compared with normal counterparts, CypA expression was increased in the idiopathic pulmonary fibrosis sarcoidosis patients [14]. Further, the serum level of CypA from pulmonary hypertension patients is closely related with the production of circulating cytokines/chemokines and growth factors [36]. In this study, by 2-DE-based proteomic approaches, we showed that CypA was overexpressed in OSF tissues. Altered expression of CypA was further verified by western blot, Q-PCR as well as IHC staining with clinical samples. More importantly, we demonstrated that CypA expression was increased in later OSF stages than early stages. Further, OSCC tissues showed an even higher average CypA expression level than that in OSF tissues. Enhanced expression of CypA in our data is in line with previous study, and suggested that CypA might be a potential biomarker of OSF.

Recently, accumulating studies showed a pro-proliferative role of CypA [32, 37, 38]. It is reported that CypA promoted the proliferation of vascular smooth muscle cells in a Rho-kinase-dependent secreted manner [39]. In current data, we found that exogenously expression of CypA facilitated proliferation of both primary fibroblast cells and IMR 90 cells. Similarly, inhibition of CypA by siRNAs or chemical antagonist induced marked apoptosis in these cells. These findings suggest that CypA is a key regulator of fibroblast viability, and contribute to OSF development probably via promoting fibroblast cell proliferation.

By bioinformatics analyses, several key factors involved in regulation of cell proliferation and apoptosis were identified in CypA PPI network, including AKT [40], ERK [41] and Caspase 3 [42]. It is previously reported that extracellular CypA was capable to stimulate proliferation in endothelial cells and vascular smooth muscle cell by activating AKT and ERK [43]. In this study, we showed that intracellular CypA could also activated AKT and ERK, suggesting a distinct mechanism. Further, we also demonstrated that knockdown of CypA by siRNAs induced notable activation of Caspase 3 and severe decrease in mitochondrial membrane potential. It is well established that loss of mitochondrial membrane potential is companied with a series of pro-apoptotic events, including release of cytochrome c, activation of Bax and cleavage of Caspase 3 [44]. Therefore, our data suggested that mitochondrial apoptosis pathway is probably involved in loss of CypA-induced cell death.

## CONCLUSION

In this study, CypA is found to be up-regulated in OSF tissues by 2-DE-based proteomic profiling. Immunostaining using clinical samples demonstrates that expression CypA is associated with several clinical indexes. Function study shows that modulation of CypA alters the proliferative and apoptotic rate of fibroblast cells. Mechanistic study revealed that overexpression of CypA increases the phosphorylation of both AKT and ERK, while loss CypA activates Caspase 3 and reduces the mitochondrial membrane potential. The current study highlights a pro-proliferative role of CypA in fibroblast, and suggests that CypA might be a potential biomarker and therapeutic target in oral submucous fibrosis.

## MATERIALS AND METHODS

### Cell culture and transfection

Human lung fibroblasts IMR90 was purchased from ATCC and the primary oral fibroblasts was separated from human normal tissues. Cells were cultured in Dulbecco's Minimum Essential Medium (Gibco) with 10% fetal bovine serum (Gibco, 37°C, 5% CO_2_). At the intensity of 70%-80%, cells were transfected with CypA specific siRNA or human CypA overexpression plasmid. SiRNA sequence was used as previously reported; siCypA1: 5-CAA GAU GAC UAA UGU CAAA-3; siCypA2: 5- CUU CUU GCU GGU CUU GCC AUU-3; siCypA3: 5-GCU UUA GGC UGU AGG UCAA-3; siNC: 5- GAA CUG AUG ACA GGG AGGC-3 [45, 46]. Transfections were conducted according to the manufacturer's introduction.

### Clinical samples

The clinical samples used in this study were collected from Xiangya Stomatological Hospital, Central South University and West China Hospital of Stomatology, Sichuan University with informed consent from patients. No preoperative treatment was performed in prior to sampling. Clinicopathologic stages of the OSF and OSCC samples were determined by two pathologists. This study was approved by the Institutional Ethics Committee of Sichuan University.

### 2-DE

100 mg of tissue sample containing both epithelium and submucosa were grounded in liquid nitrogen and lysed in lysis buffer (7M urea, 2 M thiourea, 4% CHAPS, 2 mM tributylphosphine, 2 mM PMSF, 1 mg/ml DNase I, 0.2 mg/ml RNase A). Samples were incubated on ice for extra 30 min with occasionally vortex, and then centrifuged at 14000 RPM for 1h at 4°C. The supernatant was further precipitated with ice cold acetone/trichloroacetic acid (volume 1:4), and the pellet was dissolved in rehydration buffer (7 M urea, 2 M thiourea, 4% CHAPS, 2 mM tributylphosphine, 0.2% ampholytes). After rehydration for 16 h, IEF was performed by ReadyStripTM IPG strips (17cm, pH 3–10 non-linear; Bio-Rad) on an IEF system (Bio-Rad). After IEF and 3dwater cleaning, the strips were equilibrated with DTT (0.2g/10ml) and IAM (0.25g/10ml) in the balance buffer (6M urea, 2% SDS, 0.375M Tris-HCl pH=8.8, 20% glycerol), each for 15mins. Then, strips were transferred to 12% SDS-PAGE by PROTEAN II xi Cell system (Bio-Rad) and the protein spots were visualized by coomassie brilliant staining. The SDS-PAGE gel images were obtained and analyzed by PDQuest 7.1 software (Bio-Rad). The image was normalized by the ratio of one spot to the total spot, and spot intensity was quantified by calculating the spot volume and OD value. These spots with at least 2-fold (t-test, p<0.05) or with the occurrence more than 3 times were selected for mass spectrum identification.

### In gel digestion

Spots for mass spectrum identification were obtained and digested by mass spectrometry grade trypsin gold (Promega, Madison, WI) following the manufacturer's introduction. Briefly, the spots were cut out of the gel (1-2mm diameters) and de-stained with 100 mM NH_4_HCO_3_, 50% acetonitrile at 37°C for 30 min. after totally de-stained, the gel spots were dehydrated with 100% acetonitrile and air drying for 10 mins, and then pre-incubated with 10-20μl trypsin solution (10 ng/μL) for 1 h. Before incubated overnight at 37°C, the gels were covered with digestion buffer (40 mM NH_4_HCO_3_, 10% acetonitrile). After 16h digestion, the peptides were extracted and dried in a SpeedVac concentrator (Thermo Scientific) at 4 °C. The samples were ready for mass spectrum identification.

### Mass spectrum analysis

The pellets were diluted in 50% acetonitrile with 0.2% TFA and 2.5 μl of the peptide dilution was tipped on the ground steel MALDI target. After air-dry, equal volume of 2.5mg/ml cyano-4-hydroxycinnamic acid matrix covered on the peptide and allowed to dry. The MS/MS was performed on Micro-mass Q-TOF or ESI Premier mass spectrometer (Waters, Manchester, UK). The mass spectrum was calibrated and the automatic scan rate was 1s with an inter scan delay of 0.02s. Spectra were accumulated between 900-3500 m/z and 10 parent ions of maximum intensity were selected for MS/MS analysis. The MS/MS analyzed data were processed with MASCOT (Version 2.2; Matrix Science, London, UK) against the Swiss-prot protein sequence database. The MS/MS search parameter set as follows: species, homosapien; enzyme, trypsin; allowance of up to one missed cleavage peptide; mass tolerance, 0.1 Da; MS/MS tolerance, 0.05 Da; fixed modifications of cysteine, carbamidomethylation; variable modifications of methionine, oxidation; justification threshold, p<0.05. The individual MS/MS spectrum with statistical significance was acceptable and proteins with highest MASCOT score were selected.

### Bioinformatics analysis

The protein-protein interaction (PPI) network was conducted based on the identified proteins, and biological evidence was collected from PrePPI [47]. And then, network associated proteins were classified into 10 groups according to the GO (Gene Ontology) Annotation clustering. The network group analysis was conducted via DAVID database and each functional group contains at least 100 proteins (http://david.abcc.ncifcrf.gov/) [48]. The proliferation and apoptosis associated subnetworks were identified. Cytoscape was used to integrate the unified conceptual framework of PPI network [49, 50]. Pathway annotations (derived from Kyoto encyclopedia of genes and genomes) and GO associated biological process were used for functional categories analysis. The functional annotations analysis was conducted via gene ontology tool (GOTERMCCALL) and proteins with p<0.05 (Fisher's exact test) significance would be collected in the list.

### Q-PCR

Total RNA of OSF and normal tissues was isolated by TRIzol reagent (Invitrogen) and reverse transcript to cDNA with 1μg RNA in a volume of 20 μl by ExScript^™^ reagent kit (TaKaRa, Dalian, China) according to the manufacturer's instructions. The primers were designed by Primer premier 5 and the detailed sequences were as follows: CypA: Sense: 5′-CAAGGTCCCAAAGACAGCAGA-3′, Antisense: 5′-AAG ATG CCA GGA CCC GTA TGC-3′; actin: Ssense: 5′-CAC GAT GGA GGG GCC GGA CTC ATC-3′ Antisense: 5′-TAA AGA CCT CTA TGC CAA CAC AGT-3′. Q-PCR was performed on a 7300 real-time PCR systems (Applied Biosystems). The Q-PCR was conducted following the standard procedure of SYBR Premix Ex Taq II kit (Takara).

### Western blot

Proteins were extracted in RIPA buffer (50 mM Tris base, 1.0 mM EDTA, 150 mM NaCl, 0.1% SDS, 1% Triton X-100, 1% sodium deoxycholate, 1% cocktail) and quantified by coomassie brilliant G-250 (Bio-Rad). Samples were separated and then transferred to PVDF membranes (the separation time was depending on the location of bromophenol blue in the gel). The membranes were blocked with 5% skim milk in TBST for 1h at 37°C and probed with primary antibody overnight at 4°C. After washing with TBST and membranes were incubated with secondary antibody (1:5000dilution; Santa Cruz Biotechnology) conjugated to horseradish peroxidase for 1h at 37°C. Finally, the proteins were visualized by chemiluminescence reagents (Amersham Biosciences) and actin act as internal control. CypA: #2175; Actin: SC-69879; Annexin A2: PA5-14317; ERK: #9107S; p-ERK: #4370S; AKT: #4691S; p-AKT: #38449; Active caspase 3: #9661.

### Cell proliferation assays

Cells were transfected with siRNA or plasmid. After 24h, cells were collected and allowed to grow at an intensity of 5×10^3^ cell per well. The cell was constantly cultured in 5% CO_2_ at 37°C for 48h, 72h, and 96h, and then treated with CCK8 for extra 1.5h culturing. The proliferation ability was analyzed according to the OD450nm.

For BrdU labeling assay, cells were transfected with siRNA or plasmid, and cultured for 72h. Cells were incubated with 30mM BrdU (Sigma) for another 12h and detected by BrdU fluorescent kit (KeyGEN).

### Apoptosis detection assays

The TUNEL assay was performed according to the manufacturer's introduction (Promega) and the TUNEL positive cell was counted under the fluorescence microscope (Olympus). Briefly, siCypA treated cells were seeded on the glass slide and fixed with 2% formalin for 15 min. After three times washing and 0.03% H_2_O_2_ treatment, cells were permeabilized with 0.1% TrionX-100 for 2 min at 4°C. Finally, cells were equilibrated and incubated with rTdT buffer. The nucleuses with green fluorescence under the microscope were considered as apoptosis positive.

FCM assay was conducted as the standard procedures (KeyGEN). Briefly, FCM assay was conducted as follows: cells were treated and collected; after washed with PBS for 3 times, cells were re-suspended in 500μL binding buffer and stained with PI/Annexin V for 15mins in the dark; the apoptotic cells were counted by a FCM machine (Bruker).

PI staining was conducted as several steps. Firstly, cells were interfered with siCypA and then collected; secondly, re-suspended in the PBS buffer with 0.1%TritionX-100; thirdly the cells were counted and keep the cell density at 10^6^/ml; finally the cells were stained with PI (0.5 μg/ml and RNase 1μg/ml) for 10 min and collecting the cells for microscopic examination.

JC-1 staining was conducted as the suggested protocol. The red and green staining intensity was detected via a fluorescence microplate reader (Thermo).

### Immunohistochemical analysis and clinical tissues

Masson staining was conducted according to the standard procedure (KeyGEN). CypA was expressed both in the nucleus and cytoplasm, five sections of each tissue were randomly selected and cells with positive staining were counted. The detailed procedure of IHC staining was described previously by Wang, Z., et al. [51], with some slight modifications in our experiment. Two blind scoring experiments were conducted according to the following two rules: 1) the intensity of staining corresponding to score 1; negative staining (0), mild staining (1), moderate staining (2), and strong staining (3). 2) the coverage of the positive staining in the whole sense corresponding to score 2; 5% (0), 6–25% (1), 26–50% (2), 51–75% (3), and 75%-100% (4). The total score of the staining intensity was calculated as score 1 × score 2 and the scores were used to do statistical analyzing. The average expression level of CypA in each tissue was classified into four degrees: negative (-, 0), weakly positive (+, 1-3), positive (++, 4-7), strongly positive (+++, 8-12).

### Statistical analysis

The statistical results are presented as mean ± standard deviation (S.D.) and all of the statistical analyses were repeated at least three times. ANOVA test was used to analyze the CypA expression level between different stages of OSF; Mann-Whitney test was used to analyze whether the different expression level; Spearman correlation study was used to analyze the relation between CypA expression level and OSF occurrence; Least square method was used to analyze the relation between CypA expression level and progression of different stage of OSF; Pearson correlation test was used to analyze the correlations between CypA and Annexin A2; Further, we calculated the *odds ratio* (*OR*) to validate the carcinogenesis rate of OSF. The mean values were compared by student's test and the significance was defined as *p<0.05, **p<0.01, ***p<0.001. All of the statistical analysis were conducted by SPSS 19.0.

## SUPPLEMENTARY FIGURES


