# Supplementary material for: Proteomic identification of cyclophilin A as a potential biomarker and therapeutic target in oral submucous fibrosis
Source: Oncotarget. 2016 Aug 12;7(37):60348–65. doi: 10.18632/oncotarget.11254 (PMC5312388; doi:10.18632/oncotarget.11254)
Supplement: Supplementary file 1 [file oncotarget-07-60348-s001.pdf]

Proteomic identification of cyclophilin A as a potential biomarker and therapeutic target in oral submucous fibrosis

SUPPLEMENTARY FIGURES

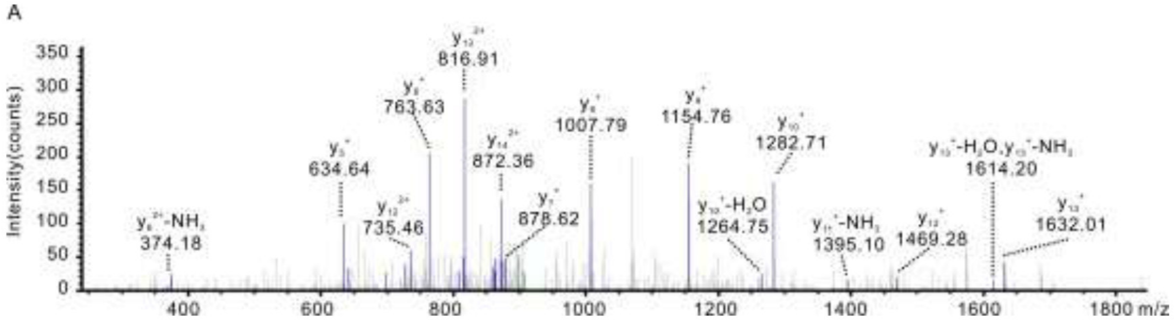

Supplementary Figure S1: Representative MS/MS data of CypA. MS/MS results corresponding to the CypA-derived peptide SIYGEKFEDE.

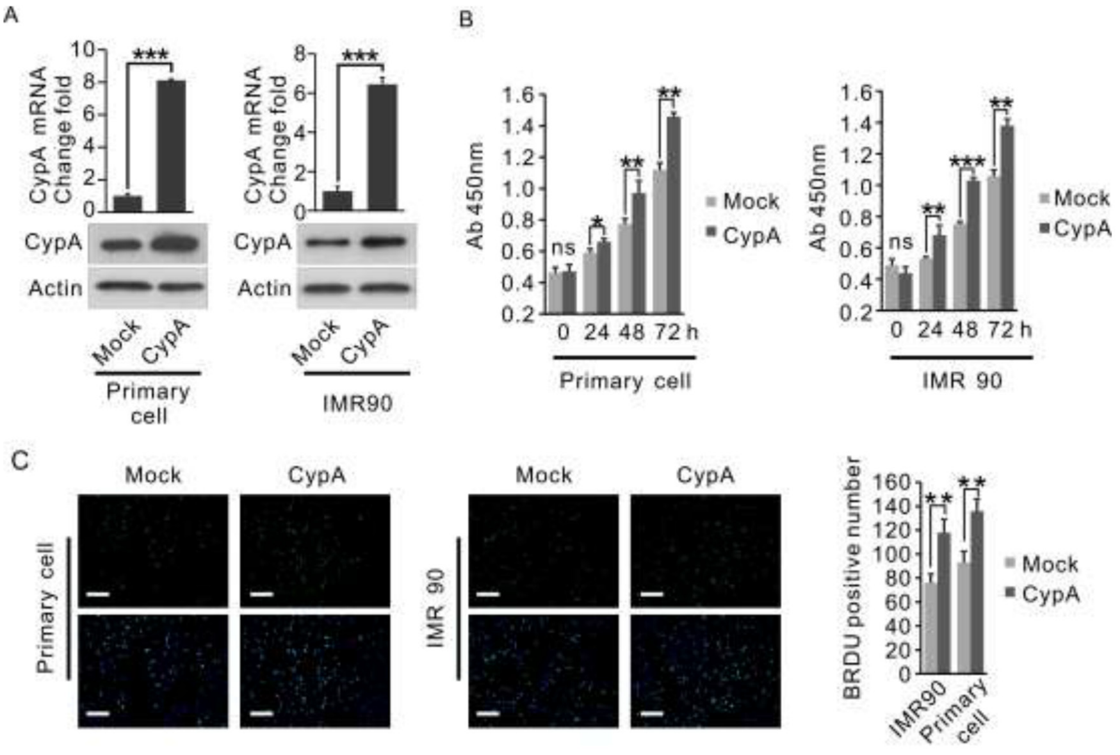

Supplementary Figure S2: Overexpression of CypA promotes fibroblast proliferation. A. IMR90 and primary oral fibroblast were transfected with transfected mock or CypA expression vector. Expression of CypA was examined by Q-PCR and western blot. **B.** IMR90 and primary oral fibroblast were transfected with transfected mock or CypA expression vector. Proliferation of IMR90 and primary oral fibroblast cells was examined by CCK8 assay. **C.** IMR90 and primary oral fibroblast were transfected with transfected mock or CypA expression vector. Proliferation of IMR90 and primary oral fibroblast cells was examined by BrdU assay. T-test, $p<0.001$ ***, $p<0.01$ **, $p<0.05$ *.
